# Analyzing transfer learning impact in biomedical cross-lingual named entity recognition and normalization

**DOI:** 10.1186/s12859-021-04247-9

**Published:** 2021-12-17

**Authors:** Renzo M. Rivera-Zavala, Paloma Martínez

**Affiliations:** Computer Science Department, University Carlos III of Madrid, Leganes, Madrid, Spain

**Keywords:** Natural language processing, Clinical texts, Deep learning, Contextual information

## Abstract

**Background:**

The volume of biomedical literature and clinical data is growing at an exponential rate. Therefore, efficient access to data described in unstructured biomedical texts is a crucial task for the biomedical industry and research. Named Entity Recognition (NER) is the first step for information and knowledge acquisition when we deal with unstructured texts. Recent NER approaches use contextualized word representations as input for a downstream classification task. However, distributed word vectors (embeddings) are very limited in Spanish and even more for the biomedical domain.

**Methods:**

In this work, we develop several biomedical Spanish word representations, and we introduce two Deep Learning approaches for pharmaceutical, chemical, and other biomedical entities recognition in Spanish clinical case texts and biomedical texts, one based on a Bi-STM-CRF model and the other on a BERT-based architecture.

**Results:**

Several Spanish biomedical embeddigns together with the two deep learning models were evaluated on the PharmaCoNER and CORD-19 datasets. The PharmaCoNER dataset is composed of a set of Spanish clinical cases annotated with drugs, chemical compounds and pharmacological substances; our extended Bi-LSTM-CRF model obtains an F-score of 85.24% on entity identification and classification and the BERT model obtains an F-score of 88.80% . For the entity normalization task, the extended Bi-LSTM-CRF model achieves an F-score of 72.85% and the BERT model achieves 79.97%. The CORD-19 dataset consists of scholarly articles written in English annotated with biomedical concepts such as disorder, species, chemical or drugs, gene and protein, enzyme and anatomy. Bi-LSTM-CRF model and BERT model obtain an F-measure of 78.23% and 78.86% on entity identification and classification, respectively on the CORD-19 dataset.

**Conclusion:**

These results prove that deep learning models with in-domain knowledge learned from large-scale datasets highly improve named entity recognition performance. Moreover, contextualized representations help to understand complexities and ambiguity inherent to biomedical texts. Embeddings based on word, concepts, senses, etc. other than those for English are required to improve NER tasks in other languages.

## Background

Efficient information extraction off biomedical data described in scientific articles, clinical narrative, or e-health reports is a growing interest in biomedical industry, research, and so forth. In this context, improved biomedical name mentions identification in the biomedical texts is a crucial step downstream tasks such as drug and protein interactions, chemical compounds, adverse drug reactions, among others. Named Entity Recognition (NER) is one of the fundamental tasks of biomedical text processing, intending to automatically extract and identify mentions of entities of interest in running text, typically through their mention boundary or by classifying tokens to match specific entity mentions. Traditionally, there are three phases in recognizing concepts in texts: (1) to identify the limits of the term or phrase that represents the concept in the text (char offsets in the text), (2) to classify the term or phrase on a class (for instance, drug, disease, body part, etc.) and (3) to normalize the concept by assigning it an identifier in a specific domain resource such as UMLS [1]. The existing biomedical NER methods can be classified into: dictionary-based methods, which are based on the use of existing domain knowledge dictionaries limited by its size, spelling errors, the use of synonyms, and the constant growth of vocabulary. Rule-based methods and Machine Learning methods usually depend on the engineering of syntactic and semantic features as well as specific language and domain features that are learned from large collections of text or built from scratch. More recently, deep learning approaches have emerged due to the availability of myriad data from different sources (scientific literature, social media, clinical texts, etc.).

The NER task has been accomplished by three types of methods. Dictionary-based methods require having specific resources integrating terminology such METAMAP tool [2] that includes UMLS [1] and recognizes mentions of medical concepts. With the availability of annotated corpora, machine learning supervised approaches have widely used in entity recognition. One of the most effective methods is Conditional Random Fields (CRF) [3] since CRF is one of the most reliable sequence labeling methods. Different challenges have been held to foster research in NER, for example, eHealth CLEF, SEMEVAL and TAC, among others. In the special case of drugs, DDIExtraction 2011 [4] and DDIExtraction 2013 [5] were specifically designed to recognize pharmacological entities and drug-drug interactions (DDI) in Medline abstracts and DrugBank technical records both in English. In these shared tasks the best result reported for NER using four types of pharmacological substances (generic drug names, branded drug names, drug group names and active substances not approved for human use) was F1 of 71.5% (by a system based on CRF algorithm). For DDI identification and classification in four classes (advice, mechanism, effect and int) the best result was 65.1% (system bases on a combination of kernels). Most of the participating systems were built on support vector machines (SVM). In general, approaches based on non-linear kernels methods achieved better results than linear SVMs.

More recently, deep learning methods started to obtain better results in NER based on the use of pre-trained models (word embeddings) obtained from a huge volume of unlabelled texts (scientific literature, social media texts, Wikipedia, among others). Word embeddings have been evolving from static representations that do not model the dynamic nature of words to contextualized representations that allow word embeddings to adapt to the context it appears, see [6] for a detailed description of embeddings. Pre-trained models may be useful for analyzing texts if these texts are similar to what they were trained on. When texts are from a different domain we will need to fine-tune a pre-trained model to fit our data or task. This is much more efficient than training a whole model from scratch because it is too time and resources consuming task. With a limited set of examples systems can get high performance in downstream tasks. See [7] for a survey of embeddings in clinical natural language processing. The new challenge, PharmacoNER 2019 [8] was focused on recognizing and normalizing pharmacological substances in Spanish clinical cases. In the current stream of deep learning approaches, participating systems mostly included those architectures. The baseline defined for PharmacoNER was based on vocabulary transfer using a LSTM model with Glove embeddings trained from SBWC and the medical word embeddings for Spanish [9] that achieved a high F1 of 0.82% (ranked 16 out of 22) in NER. The first ranked system [10] was based on a pipeline composed of a BERT (Bidirectional Encoder Representations from Transformers) for NER and a Bi-LSTM for concept indexing achieving an F1-score of 91.5% on NER and 83.9% on concept indexing. The third-ranked system [11] was based on a Bi-LSTM-CRF tagger with FLAIR contextualized embeddings obtaining a result of 89.76% F1-score using pre-trained embeddings and up to 90.5% using specialized ones. The second-ranked system [12] implemented a traditional knowledge-based approach based on dictionaries, particularly the SNOMED-CT medical ontology [13] together with a set of 104 contextual regexp patterns to tackle ambiguity (an important issue especially for abbreviations) and surprisingly this system obtained an F1-Score of 91% in NER and 91.6% in concept indexing (top system). This reveals that resource-based approaches have a lot to say yet. Other deep learning works have also demonstrated state-of-the-art performance for English [14–16] texts by automatically learning relevant patterns from corpora, which allows language and domain independence. Weber [17] described a set of experiments with a NER tool called HUNER that incorporates a fully trained LSTM-CRF model using 34 different corpora for five entity types that outperform the state-of-the-art tools CnormPlus and tmChem by 5–13 pp for chemicals, species and gene on CRAFT corpus [18]. However, concerning the generation of domain-based pre-trained models until now, to the best of our knowledge, there is only one work that addresses the generation of Spanish biomedical word embeddings [9, 19].

In this paper, we propose two deep learning approaches to face the recognition of pharmacological and chemical entities in Spanish texts. The approaches are evaluated using the Spanish biomedical PharmaCoNER and English biomedical CORD-19 datasets. Our main goal is to evaluate the performance impact of cross-domain (general and biomedical domain) and cross-language (Spanish and English) pre-trained embeddings models. Firstly, for entity identification and classification, we implemented two bidirectional Long Short Memory (Bi-LSTM) layers with a CRF layer based on the NeuroNER model proposed in [20]. Specifically, we have extended the NeuroNER [20] architecture by adding context information to token-level representation, such as Part-of-Speech (PoS) tags and overlapping or nested entities. Moreover, in this work, we use several pre-trained word embedding models: (i) a word2vec model (Spanish Billion Word Embeddings [21]), which was trained on the 2014 dump of Wikipedia, (ii) pre-trained word2vec model of word embeddings trained with PubMed and PMC articles, (iii) Scielo and Wikipedia cased pre-trained model based on the FastText implementation, (iv) a sense-disambiguation embedding model [22], where different word senses are represented with different sense vectors and trained from scratch embedding models (v) the FastText-SBC model trained on the FastText implementation and (vi) the SNOMED-SBC model based on the FastText-SBC replacing concepts with their unique SNOMED-CT [13] identifier. Finally, we implemented the Bidirectional Encoder Representations for Transformers (BERT) model with fine-tuning using BERT pre-trained general domain models and a trained from scratch biomedical model. For concept indexing based on the output of offset recognition and entity classification, we applied a full-text search and a fuzzy matching approach on the SNOMED-CT Spanish Edition dictionary to obtain the corresponding index to normalize the concept.

## Results

We evaluate our deep learning models using the train, validation and test datasets provided by the task organizers of the PharmaCoNER Shared Task [8]. The PharmaCoNER task considers two subtasks. Subtask 1 considers offset recognition and entity classification of pharmacological substances, compounds, and proteins. Subtask 2 considers concept indexing where for each entity, the list of unique SNOMED concept identifiers must be generated. We apply the standard measures precision, recall and F1-score to evaluate the performance of our approaches. These metrics are also used in the PharmaCoNER task. A detailed description of the evaluation can be found in [23].

Moreover, we evaluate our deep learning models on the train, validation and test subsets of the CORD-19 dataset [24]. F-measure is used as the primary metric where true positives are entities that match with the gold standard annotations boundaries and entity type.

### Offset detection and entity classification

The NER task is addressed as a sequence labeling task. For NER we tested different configurations with various pre-trained word representation models.

#### Bi-LSTM CRF model: extended NeuroNER

For our Bi-LSTM CRF model we test various pre-trained and trained from the scratch word embeddings models (see Table 21). Table 1 describes our different experiment configurations for the PharmaCoNER datasets with Spanish general domain (W2V-SBWC and FastText-SBWC), English general domain (FastText 2M), Spanish biomedical domain (FastText-SBC and SNOMED-SBC) and English biomedical domain (PubdMed and PMC) embeddings. Each configuration for all evaluations was executed up to 5 times and we kept the best result obtained (85.75) as shown in Table 2. Table 2 compares the different results obtained in 5 runs for Extended NeuroNER using FastText-SBC + Reddit embedding models.

**Table 1:** System hyperparameters for each PharmaCoNER run

Parameter	Run 1	Run 2	Run 3	Run 4
Sense-disambiguation embedding dimension	128	128	128	128
Pre-trained word embeddings	FastText-SBC + Reddit	W2V-SBWC + Reddit	FastText-SBWC + Reddit	SNOMED-SBC + Reddit
Word embeddings dimension	300	300	300	300
Character embedding dimension	50	50	50	50
Hidden layers dimension (for each LSTM)	100	100	100	100
Learning method	SGD	SGD	SGD	SGD
Dropout rate	0.5	0.5	0.5	0.5
Learning rate	0.005	0.005	0.005	0.005
Epochs	100	100	100	100

**Table 2:** Extended NeuroNER with FastText-SBC + Reddit embedding models runs results according to Table 1 configurations

Experiment	Precision (%)	Recall (%)	F-score (%)
Run 1	88.19	82.61	85.31
Run 2	87.77	**83.65**	85.66
Run 3	**90.0**	80.9	85.21
Run 4	89.13	82.61	**85.75**
Run 5	88.03	82.96	85.42

Bold values are the best results for Precision (P), Recall (R) and F-score

Table 4 shows a comparison of the different pre-trained models on the PharmaCoNER validation dataset where we want to highlight that domain-specific and word embeddings outperform general domain models by almost 5 points, Moreover, language-specific word embeddings outperform cross-lingual models by almost 4 points. Furthermore, lower performance of general domain and cross-lingual word embeddings models can be related to recall performance; this can be interpreted as many out-of-vocabulary words. For the test dataset, we applied our best system configuration FastText-SBC + Reddit (see Table 4) obtaining an F-score of 85.24% for offset detection and entity classification. Furthermore, Table 3 shows the classification results obtained by our best system configuration for offset detection and entity classification with a micro average of 88.10% for PharmaCoNER valid dataset.

**Table 3:** Extended NeuroNER results for each entity on PharmaCoNER valid dataset

Entity	Precision (%)	Recall (%)	F-score (%)
Normalizables	92.38	**86.41**	**89.29**
No_Normalizables	0.00	0.00	0.00
Proteins	**93.29**	85.35	89.14
Unclear	87.80	70.59	78.26
Micro-average	91.75	84.74	88.10

Bold values are the best results for Precision (P), Recall (R) and F-score

**Table 4:** Results for Extended NeuroNER entity classification using combinations of embeddings models on PharmaCoNER test dataset

Experiment	Embedding model	Precision (%)	Recall (%)	F-score (%)
Run 4	SNOMED-SBC + Reddit	83.52	74.97	79.02
Run 2	W2V-SBWC + Reddit	83.85	75.75	79.60
Run 3	FastText-SBWC + Reddit	84.70	77.31	80.84
Run 1	FastText-SBC + Reddit	**89.13**	82.61	**85.75**
Out of task	Scielo+Wiki cased + Reddit	86.69	**82.72**	84.66
Out of task	PubMed and PMC + Reddit	87.23	76.98	81.79
Out of task	FastText 2M + Reddit	84.04	77.55	80.67

Bold values are the best results for Precision (P), Recall (R) and F-score

Moreover, we compared our best system configuration (FastText-SBC + Reddit) with the baseline NeuroNER model (without sense embeddings and BMEWO-V format encoding) using the same FastText-SBC embedding and configuration. Table 5 shows that our extended system outperforms the NeuroNER base system, which has proven that sense embeddings and BMEWO-V format to be an additional source of information to deal with ambiguity and nested entities (see “Methods” section for detail about BMEWO-V format). Furthermore, the use of domain-specific word embeddings highly improves performance as is shown in Table 4.

**Table 5:** Baseline comparison for entity classification on PharmaCoNER test dataset

System	Precision (%)	Recall (%)	F-score (%)
NeuroNER	86.38	82.07	84.16
Extended NeuroNER	**89.13**	**82.61**	**85.75**

Bold values are the best results for Precision (P), Recall (R) and F-score

Furthermore, we tested the FastText-2M English general domain and the Pubmed and PMC English domain-specific non-contextualized pre-trained embeddings models (more details in Table 21) on the CORD-19 dataset. Table 6 describes our different experiment configurations for the CORD-19 dataset.

**Table 6:** System hyperparameters for CORD-19 experiments

Parameter	Experiment 1	Experiment 2
Sense-disambiguation embedding dimension	128	128
Pre-trained word embeddings	Pubmed and PMC + Reddit	FastText 2M + Reddit
Word embeddings dimension	300	300
Character embedding dimension	50	50
Hidden layers dimension (for each LSTM)	100	100
Learning method	SGD	SGD
Dropout rate	0.5	0.5
Learning rate	0.005	0.005
Epochs	100	100

**Table 7:** Extended NeuroNER results for each entity on CORD-19 test dataset

Entity	Precision (%)	Recall (%)	F-score (%)
Chemical or Drug	81.86	**83.52**	82.68
Disorder	**85.73**	80.77	**83.17**
Protein or Gene	63.81	49.40	55.69
Micro-average	81.17	75.49	78.23

Bold values are the best results for Precision (P), Recall (R) and F-score

In Table 8, we compare the FastText-2M model trained on English general domain texts and Pubmed and PMC model trained on English biomedical texts (more details in Table 22), both tested on the CORD-19 test dataset. As shown in Table 8, domain-specific models outperform general domain models by almost 3 points, obtaining an F-score of 78.23% for offset detection and entity classification. Table 7 shows the classification results obtained by our best system configuration for offset detection and entity classification with a micro average F-score of 78.23% for the CORD-19 test dataset. Classification results on Protein/Gene are lower than other entities type mainly due to ambiguity and short named entity detection.

**Table 8:** Extended NeuroNER results for entity classification on CORD-19 test dataset

Embedding model	Precision (%)	Recall (%)	F-score (%)
Pubmed and PMC + Reddit	**81.17**	**75.49**	**78.23**
FastText 2M + Reddit	77.77	73.71	75.69

Bold values are the best results for Precision (P), Recall (R) and F-score

#### Multi-layer bidirectional transformer encoder: BERT

Additionally, we compare the different contextualized word models using the BERT implementation on the PharmaCoNER and CORD-19 test dataset with 12 transformer layers, 768-hidden, 12-heads, 110M parameters trained on each pre-trained model and fine-tuned for NER using a single output layer based on the representations from its last layer to compute only token level BIOES-V probabilities. BERT directly learns WordPiece embeddings during pre-training and fine-tuning steps. BERT provides subword representations. Subwords are used for representing both the input text and the output tokens. Out of vocabulary words are sliced into multiple subwords, even reaching character subwords if needed. However, subwords representations do not necessarily fit with word representation in a given context.

We compare the different general domain English pre-trained (bert-base-multilingual-cased and BETO cased) and domain-specific English pre-trained (SBC-BERT) contextualized word embeddings. As shown in Table 9 domain-specific word representations outperform general domain models by almost 7 points. Nonetheless, to the best of our knowledge there is no open pre-trained contextualized word biomedical Spanish model. Moreover, Table 10 shows the classification results obtained by our best model for offset detection and entity classification with a micro average F-score of 88.80% for PharmaCoNER test dataset.

**Table 9:** Results of BERT systems for entity classification on PharmaCoNER test dataset

System	Precision (%)	Recall (%)	F-score (%)
bert-base-multilingual-cased	84.01	76.91	80.23
BETO cased	84.68	79.02	81.66
**SBC-BERT**	**87.88**	**89.74**	**88.80**

Bold values are the best results for Precision (P), Recall (R) and F-score

We compare our deep learning approaches with the participating systems presented in the PharmaCoNER task. A detailed description of the evaluation and the participant systems is provided in [25]. As can be seen in Table 11, our SBC-BERT model reaches satisfactory performance, however is outperformed by other approaches adding more complex language and domain-specific features.

**Table 10:** Results of SBC-BERT system for entity classification on test PharmaCoNER dataset

Entity	Precision (%)	Recall (%)	F-score (%)
PROTEINAS	84.46	88.46	86.41
NORMALIZABLES	**91.86**	**92.02**	**91.94**
UNCLEAR	70.59	81.82	75.79
NO_NORMALIZABLES	15.38	12.5	13.79
**micro avg**	87.88	89.74	88.80

Bold values are the best results for Precision (P), Recall (R) and F-score

**Table 11:** Comparison of participant systems and ours on PharmaCoNER test dataset

Name	Precision (%)	Recall﻿ (%)	F-score﻿ (%)
xiongying [10]	**91.22**	90.87	**91.05**
FSL [12]	90.62	**91.31**	90.96
m-stoeckel [11]	90.70	89.08	90.46
CongSun [26]	88.05	89.24	88.64
SBC-BERT	87.88	89.74	88.80
Extended NeuroNER	89.13	82.61	85.75

Bold values are the best results for Precision (P), Recall (R) and F-score

Moreover, we test different contextualized word pre-trained models on the CORD-19 test dataset. As shown in Table 13, domain-specific word representations outperform general domain models by almost 5 points. Based in our experiments, we found that the use of domain-specific contextualized word representations highly improves the entity classification task. Table 12 shows the classification results obtained by our best BERT system configuration for offset detection and entity classification with a micro average of 78.86% for CORD-19 test dataset.

**Table 12:** BERT results for each entity on CORD-19 test dataset

Entity	Precision (%)	Recall (%)	F-score (%)
Chemical or Drug	**86.05**	83.71	**84.86**
Disorder	83.68	**84.37**	84.02
Protein or Gene	54.00	65.06	59.02
Micro-average	77.28	80.52	78.86

Bold values are the best results for Precision (P), Recall (R) and F-score

**Table 13:** Contextualized word models results for entity classification on CORD-19 test dataset

System	Precision (%)	Recall (%)	F-score (%)
bert-base-multilingual-cased	72.12	75.92	73.89
BioBERT Large	**77.28**	**80.52**	**78.86**

Bold values are the best results for Precision (P), Recall (R) and F-score

### Concept indexing

For concept indexing or normalization, we applied the same approach described for SNOMED-SBC model training, replacing each entity detected in the entity recognition and classification step with their unique SNOMED-CT Spanish Edition identifier. First, we applied a lowercase conversion, then we replace abbreviations with their corresponding full concept name using the Spanish Medical Abbreviation DataBase (AbreMES-DB) [27] and the SEDOM Medical Abbreviation Dictionary [28] for normalizing biomedical entities. We used the PyMedTermino library employing a two-stage search using full-text search and fuzzy search for concepts not found by partial matching. A full-text search with the Levenshtein distance algorithm [29] was applied in a first instance for concept indexing and fuzzy search with threshold using FuzzyDict implementation [14] as a second approach for concepts not found in the first instance by partial matching. Table 14 shows our result on concept indexing for PharmaCoNER test subset. We apply the standard measures precision, recall and micro-averaged F1-score to evaluate the effectiveness of our model, given as the evaluation metrics by the PharmaCoNER NER and concept indexing task. Results from the previous NER step are passed over for concept indexing. As shown in Table 14, BERT approach outperforms Extended NeuroNER mainly for the ability of BERT approach to resolve ambiguity.

**Table 14:** Results for concept indexing on PharmaCoNER test dataset

System	Precision (%)	Recall (%)	F-score (%)
SBC-BERT	**87.34**	**73.75**	**79.97**
Extended NeuroNER	84.17	64.22	72.85

Bold values are the best results for Precision (P), Recall (R) and F-score

Our results for concept indexing are low due to a large number of misspellings entities, abbreviations ambiguity, drug names where the identifier corresponds to the active substance as “durogesic” (“Duragesic”) active ingredient “fentanyl” (“fentanyl”), identifiers not existing in SNOMED CT, such as CHEBI:135810 and 373757009 and false positives, such as diseases identified as NORMALIZABLE entities and PROTEIN entities not annotated in the PharmaCoNER corpus.

## Discussion

We used different pre-trained models and investigated their effect on performance. For Extended NeuroNER, we used general and specific-domain pre-trained word embedding models, likewise we used pre-trained multi-language and language-specific models. We found that the use of a domain-specific (biomedical) and language-specific pre-trained models highly improve the NER task. In addition, to the best of our knowledge, there is no open pre-trained biomedical Spanish model for context-dependent word representations (pre-trained BERT). The base BERT model without extensions outperforms Extended NeuroNER model and other PharmaCoNER participant approaches, mainly due to its capability to deal with ambiguity problems.

We found that the text pre-processing (sentences split and tokenization) step had a significant impact on the entity offset recognition and classification mainly due out-of-vocabulary words. Additionally, we analyzed the confusion matrices for PharmaCoNER (see Table  15) and CORD-19 (see Table 16) datasets, where the leading diagonal represents correctly classified tokens (true positives and true negatives) and the cells above and below the leading diagonal misclassified tokens (false positives and false negatives). We can see for PhamarCoNER dataset that the greatest amount of missclassified tokens (269) occurs with the PROTEINAS type entity and in the same way for CORD-19 dataset the greatest amount of misclassified tokens (452) occurs with the PRGE (protein or gene) type entity. This can be attributed to a large number of abbreviations and short-length entities. Furthermore, on false positives and false negatives error analysis we found that: (i) separating words by the hyphen ‘-’ caused some errors (e.g., S-100, Alfa-Feto-Proteina). (ii) Abbreviation recognition is a difficult task due to ambiguity and length, even more for very short abbreviations (1–2 letters) due to their high level of ambiguity (e.g., CK 7, sY86, sY84, SRY, ZFY, Hb). (iii) Long entities consisting of more than five tokens are hard to identify correctly (e.g., Antigeno Prostatico Especifico, Antigeno Carcino Embrionario). (iv) Misspelling entities cause errors in concept indexing (e.g., lacticodeshidrogenasa, tenecteplasa). (v) Also, words do not present in the pre-trained models’ vocabulary are not recognized in entity offset recognition and classification.

**Table 15:**
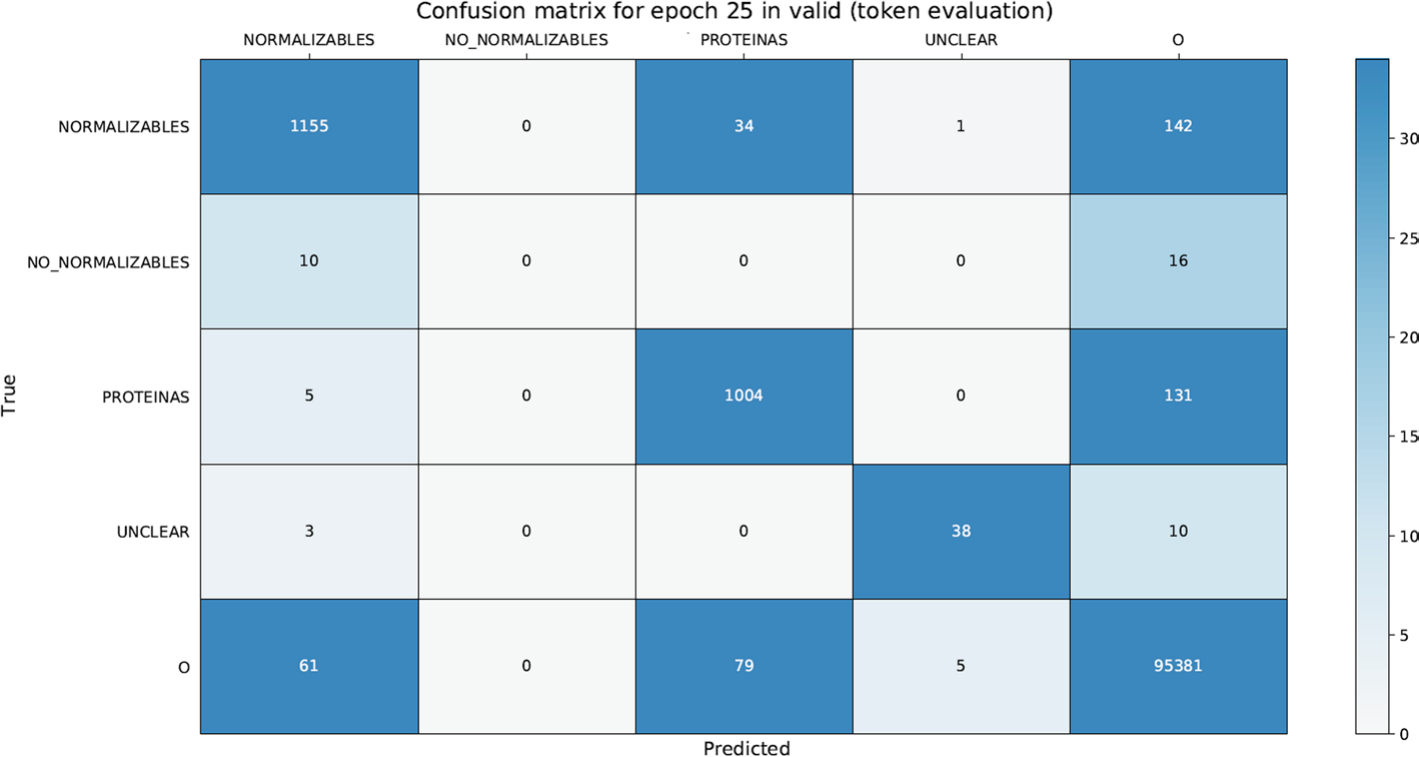
PharmaCoNER confusion matrix on test dataset for Extended NeuroNER best configuration

**Table 16:**
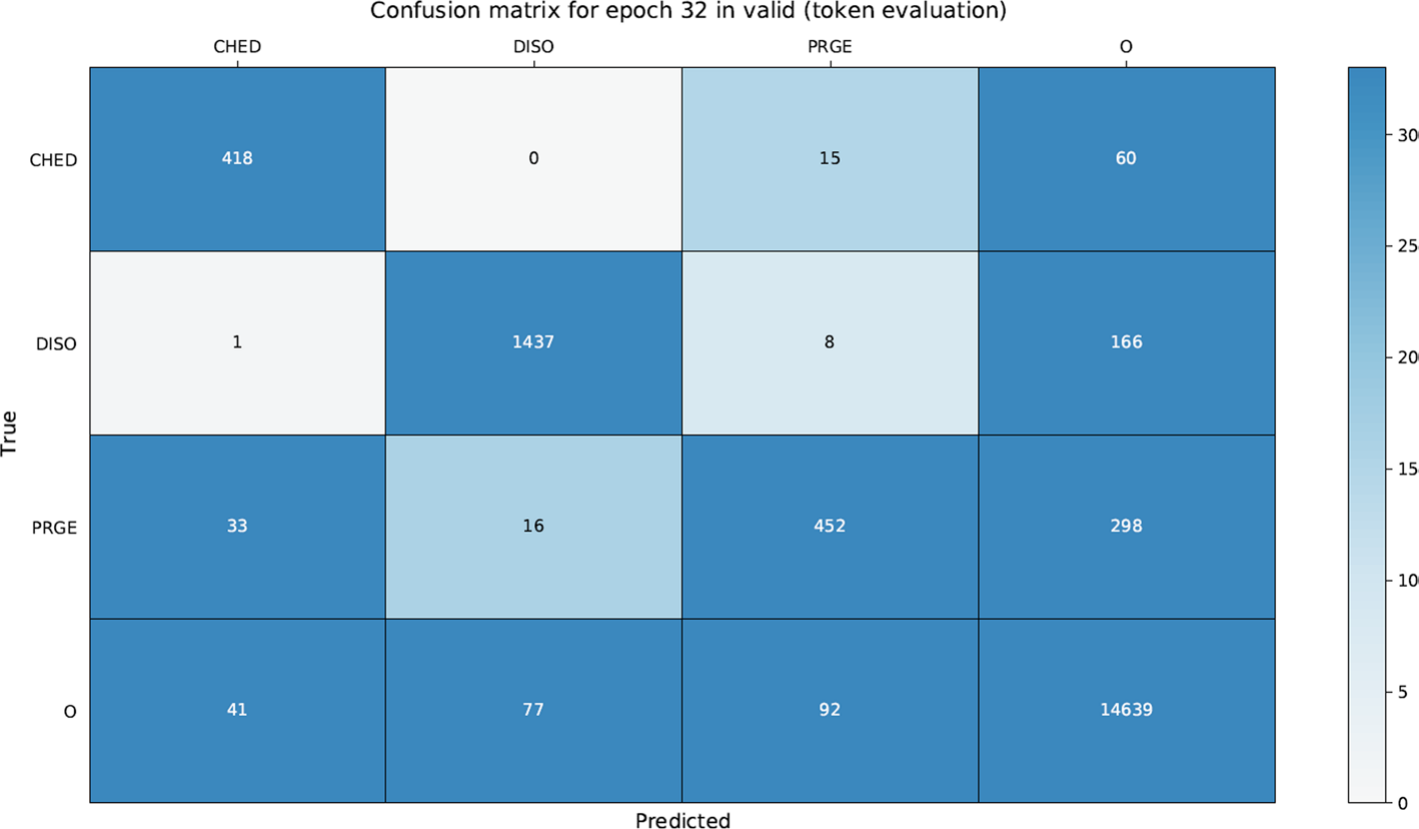
CORD-19 confusion matrix on valid dataset for Extended NeuroNER best configuration

Finally, entity recognition mistakes from offset detection and entity classification are propagated to the concept indexing task. There are about 10% errors caused by offset detection and entity classification. In addition, about 10% errors are caused by the concept indexing model. About 40% entities are abbreviations, which is difficult to find the appropriate concept from SNOMED-CT which only considers full concept name. Moreover, about 20% of entities have the same candidates in SNOMED-CT, which are not normalized entities in the shared task. This proves that shorter sentences and shorter entities are easier to process. Longer are the sentence more complex syntactic structures it carries, and tougher it is to be processed by the system.

## Conclusions

In this work, we propose a system for biomedical concept detection such as chemical compounds, drugs, disorders, chemicals, genes, and proteins in clinical narrative and biomedical texts written in Spanish and English. We address the named entity recognition task as a sequence labeling task. Our deep learning approaches only use dense vector representations features instead of hand-crafted word-based features. We proved that as in other tasks such as NER, the use of dense representation of words such as word-level, character-level, and sense no-contextualized and contextualized representations are helpful for named entity recognition. Moreover, domain and language specific embedding models outperform general domain and cross-lingual models mainly due to the non-existence of vectors for words that are not found in the vocabulary. Our approaches achieved satisfactory performance with an F-score of 85.25% for Extended NeuroNER and 88.80% for SBC-BERT. Although the BERT model outperforms the Extended NeuroNER model, the BERT model is highly expensive to train in terms of time and cost. Besides, as mentioned before out of vocabulary words are not recognized in the offset and classification step. The Extended NeuroNER and BERT models are domain-independent and could be used in other fields, although generic and domain-specific pre-trained word representations are used for this work. Moreover, new pre-trained Biomedical Spanish word embeddings (contextualized and no-contextualized) and concept embeddings have been generated for this work.

More initiatives to foster the availability of sufficiently large clinical narrative corpora in Spanish from hospitals or regional health systems are necessary. This will allow us to train embeddings of different types such as knowledge enhanced word embeddings that combine text corpora with terminology resources. Resources similar to clinical concept embeddings (cui2vec) obtained in [30] from 20 million clinical notes and 1.7 million full-text biomedical journal articles using UMLS could be useful as specialized biomedical embeddings. New approaches to extend the scope of embeddings such as [31] that use BERT to pre-trained contextualized embeddings models on structured diagnosis data from 28,490,650 patients EHR dataset to be used in disease prediction could be of great interest in clinical practice.

As future work, we plan to enhance the SNOMED-CT concept representations in concept indexing step. Furthermore, we plan to generate contextualized word representations integrating biomedical knowledge into our system such as SNOMED-CT or UMLS. The motivation would be to see whether contextualized word representations generated with biomedical knowledge can help to improve the results and provide a deep learning model for biomedical NER and concept indexing.

## Methods

In this section, we described our NER approach. Additionally, we introduce the corpora used to generate our train from the scratch contextualized and no-contextualized word representations. Furthermore, we described our deep learning approaches. We first present a deep network with a pre-processing step, a learning transfer step, then two recurrent neural network layers and the last layer with CRF classifier and a deep learning model based on a multi-layer bidirectional transformer encoder. Finally, the datasets used for training, validating, and evaluating our deep learning models performance.

### Named entity recognition

**Fig. 1:**
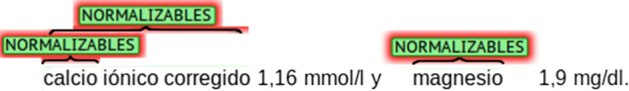
BRAT annotation example from PharmaCoNER corpus sentence where three entities are shown, two of them nested entities - “calcio iónico corregido” and “calcio”

In order to train our model, first texts must be preprocessed to create the input for the deep network. Sentences are split and tokenized using Spacy [32], an open-source library for advanced NLP with support for 26 languages. The output from the previous process is formatted to BRAT format [33]. BRAT is a standoff format where each line represents an annotation (such as entity, relation, event). We use the information from the BRAT format (see an example in Fig. 1) and then annotate each token in a sentence using the BMEWO-V extended tag encoding which is a contribution of authors. Table 17 shows an example of every possible tags for each entity type within the PharmaCoNER dataset. The BMEWO-V encoding allows us to capture information about the sequence of tokens in the sentence.

**Table 17:** 21 entity tags for BMEWO-V tag encoding on PharmaCoNER dataset where NORMALIZABLES and NO_NORMALIZABLES refer to chemical entities, PROTEINAS are proteins entities and UNCLEAR refer to tokens different from chemical or protein mentions [34]

Entity	Tags
NORMALIZABLES	B/M/E/W/V- NORMALIZABLES
NO_NORMALIZABLES	B/M/E/W/V- NO_NORMALIZABLES
PROTEINAS	B/M/E/W/V- PROTEINAS
UNCLEAR	B/M/E/W/V- UNCLEAR
Others	O

The BMEWO-V encoding distinguishes the B tag to indicate the start of an entity, the M tag representing the continuity of an entity, the E tag as the end of an entity, the W tag for indicating a single entity, and the O tag to represent other tokens that do not belong to any entity. The V tag allows representing overlapping entities. BMEWO-V is similar to other previous encodings [35]; however, we introduce the V tag to allow the representation of overlapping or nested entities which are usual phenomena in these types of texts. Additionally, we tested the BMEWO-V enconding format in previous works [16, 36]. Finally, the BRAT format is transformed into sentences annotated in the CoNLL-2003 format [37]. This is the input for our deep learning models, as is shown in Table 18.

**Table 18:** Tokens annotated for the sentence “instaurándose tratamiento con corticoides orales en forma de prednisona oral” in the ConLL-2003 format

Token	Entity	Start offset	End offset	Tag	Tag
instaurándose	Others	950	963	O	O
tratamiento	Others	964	975	O	O
con	Others	976	979	O	O
corticoides	NORMALIZABLES	980	991	B-NORMALIZABLES	W-NORMALIZABLES
orales	Others	992	998	O	O
en	Others	999	1001	O	O
forma	Others	1002	1007	O	O
de	Others	1008	1010	O	O
prednisona	NORMALIZABLES	1011	1021	B-NORMALIZABLES	W-NORMALIZABLES
oral	Others	1022	1026	O	O

This sentence has two drugs: “corticoides” and “prednisona” with B_NORMALIZABLES tag (start of entity) and W_NORMALIZABLES tag (single entity)

### Corpora

In order to generate from scratch Spanish biomedical word representations to use in this research we gathered raw biomedical Spanish text from different sources. Source corpus details are described in Table 19:

1. The Spanish Bibliographical Index in Health Sciences (IBECS) corpus [38] that collects scientific journals covering multiple fields in health sciences.
2. Scientific Electronic Library Online (SciELO) corpus [39] gathers electronic publications of complete full-text articles from scientific journals of Latin America, South Africa and Spain.
3. MedlineNLM corpus obtained from the PubMed free search engine [40].
4. The MedlinePlus corpus [41] (an online information service provided by the U.S. National Library of Medicine), consists of Health topics, Drugs and supplements, Medical Encyclopedia and Laboratory test information.
5. The UFAL corpus [42] is a collection of parallel corpora of medical and general domain texts.

**Table 19:** Biomedical Spanish corpus details

Collection\Corpus	IBECS	SciELO	MedlineNLM	MedlinePlus	UFAL
Documents	168,198	161,710	330,928	1063	265,410
Words	23,648,768	26,169,655	4,710,191	217,515	41,604,517
Unique Words	184,936	159,997	20,942	5099	198,424

**Fig. 2:**
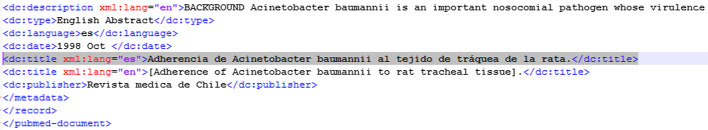
Dublin core format for biomedical corpus

All the corpora are in XML (Dublin core format) and TXT format files. XML files were processed for extract only raw text from specific XML tags such as “title” and “description” from Spanish labels, based on the Dublin Core format as shown in Fig. 2. TXT files were not processed. Raw texts from all files were compiled in a single TXT file. Texts were processed, setting all to lower, removing punctuation marks, trailing spaces and stop words and used as input to generate our word embeddings. Sentences pre-processing (split and tokenized) were made using Spacy [43], an open-source python library for advanced multi-language natural language processing.

### Bi-LSTM CRF model: extended NeuroNER

**Fig. 3:**
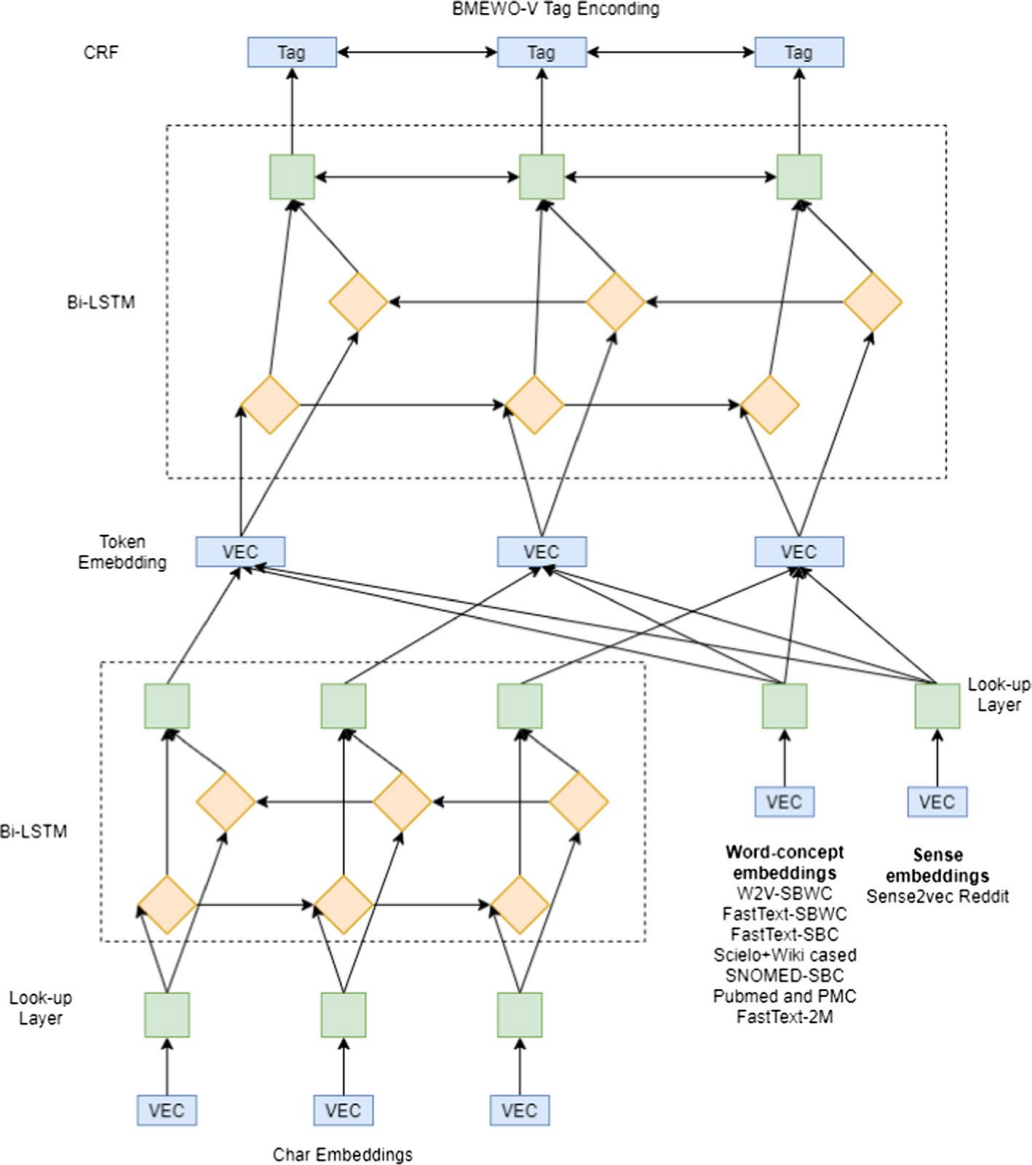
The architecture of the hybrid Bi-LSTM CRF model for named entity recognition

Our proposal involves the adaption of a NER model named NeuroNER [20] based on deep learning to identify drug and chemical mentions. The architecture of our model consists of a first Bi-LSTM layer for character embeddings. In the second layer, we concatenate the output of the first layer with the word embeddings and sense-disambiguate embeddings for the second Bi-LSTM layer. Finally, the last layer uses a CRF to obtain the most suitable labels for each token. An overview of the system architecture can be seen in Fig. 3.

To facilitate our model training, we first perform a learning transfer step. Learning transfer aims to perform a task on a dataset using knowledge learned from a previous dataset [44]. As is shown in many works, such as speech recognition [45], sentence classification [46] and Named Entity Recognition [47] transfer learning improves generalization of the model, reduces training time on the target dataset, and reduces the amount of labeled data needed to obtain high performance. We propose learning transfer as input for our model using two different pre-trained embeddings models: (i) word embeddings and (ii) sense-disambiguation embeddings. Word embedding is an approach to represent words as vectors of real numbers which have gained much popularity among the NLP community because they are able to capture syntactic and semantic information among words.

Although word embedding models are able to capture syntactic and semantic information, other linguistic information such as morphological information, orthographic transcription or POS tags are not exploited in these models. According to [48], the use of character embeddings improves learning for specific domains and is useful for morphologically rich languages (as is the case of the Spanish language). For this reason, we decided to consider the character embedding representation in our system to obtain morphological and orthographic information from words. We used a 25 features vector to represent each character. In this way, tokens in sentences are represented by their corresponding character embeddings, which are the input for our Bi-LSTM network.

In this work, we used various Spanish and English pre-trained embedding models. The Spanish Billion Words Corpora (SBWC) [21] (W2V-SBWC), which is a pre-trained word embeddings model trained on different general domain text corpora written in Spanish (such Ancora Corpus [49] and Wikipedia) using the word2vec [50] implementation. The FastText-SBWC pre-trained word embeddings model was trained on the SBWC using the FastText implementation. The Scielo+Wiki cased [51] pre-trained word embeddings model trained on biomedical domain texts from Scielo and Wikipedia using the FastText implementation. We also integrate the sense2vec [22] model, which provides multiple dense vector representations for each word based on the sense of the word. This model is able to analyze the context of a word based on the lexical and grammatical properties of words and then assigns its more adequate vector. Each word in this model is paired with its corresponding Part-of-Speech (PoS) tag. Sense2vec use the Polyglot Part-of-Speech tagger from Al-Rfou more details in [22]. We used the Reddit Vector, a pre-trained model of sense-disambiguation representation vectors presented by [22]. This model was trained on a collection of general domain comments published on Reddit (corresponding to the year 2015) written in Spanish and English. The FastText-2M [52] pre-trained English word embedding model trained with subword information on Common Crawl using the FastText implementation. Finally, the PubMed and PMC [53] pre-trained English word embedding model, trained on a combination of PubMed abstracts and full-text documents from the PMC using the word2vec skip-gram model implementation.

Furthermore, we used the FastText [54] implementation to train our own word embeddings using the Spanish Biomedical Corpora (SBC) described in section Corpora  (FastText-SBC). Moreover, we trained a concept embedding model replacing biomedical concepts in the SBC with their unique SNOMED-CT Spanish Edition identifier (SNOMED-SBC). First, we applied a lowercase conversion, then we replace abbreviations with their corresponding full concept name using the Spanish Medical Abbreviation DataBase (AbreMES-DB) [27] and the SEDOM Medical Abbreviation Dictionary [28] for normalizing biomedical entities. We used the PyMedTermino library [55] for concept indexing. We proposed two dictionary-based approaches. A full-text search with the Levenshtein distance algorithm [29] was applied in a first instance for concept indexing and fuzzy search with threshold using FuzzyDict implementation [14] as a second approach for concepts not found by partial matching. The FastText model uses a combination of various subcomponents to produce high-quality embeddings. It uses a standard CBOW or skip-gram models, with position-dependent weighting, phrase representations, and sub-word information in a combined manner. The training parameters for each model are shown in Table 20. Our pre-trained models can be found in Github [56] with the corpora sources, text pre-processing, and training information.

**Table 20:** Training parameters for embeddings models built in this work

Parameter\Model	FastText-SBC	SNOMED-SBC
Number of negatives sampled	20	20
Sampling threshold	6e$-$5	6e$-$5
Minimum number of word occurrences	10	10
Minimum length of character n-gram	3	3
Maximum length of character n-gram	6	6
Size of word vectors	300	300
Epochs	10	10
Processor	4 Intel Xeon 2.00 GHz, 8 Cores, 16 Logical Processors	4 Intel Xeon 2.00 GHz, 8 Cores, 16 Logical Processors
RAM	32 GB	32 GB
Corpus size	1 GB	1 GB
Training time	4 h	8 h

The embedding models and their parameters are summarized in Table 21.

**Table 21:** Embedding models details

Embedding model	Language	Domain	Type	Corpus size	Vocab size	Array size	Algorithm	Property
W2V-SBWC	Spanish	General	Word	1.5 billion	68k	300	Word2Vec Skip-gram BOW	Pre-trained
FastText-SBWC	Spanish	General	Word	1.5 billion	81.2k	300	FastText Skip-gram BOW	Pre-trained
FastText-SBC	Spanish	Specific (Biomedical)	Word	600 billion	91.7k	300	FastText Skip-gram BOW	Own
Scielo+Wiki cased	Spanish	Specific (Biomedical)	Word		50k	300	FastText Skip-gram BOW	Pre-trained
SNOMED-SBC	Spanish	Specific (Biomedical)	Concept	600 billion	88.1k	300	FastText Skip-gram BOW	Own
Pubmed and PMC	English	Specific (Biomedical)	Word	2 billion	400k	300	Word2Vec Skip-gram BOW	Pre-trained
FastText-2M	English	General	Word	600 billion	2 million	300	FastText Skip-gram BOW	Pre-trained
Sense2vec Reddit	English/Spanish	General	Sense	2 billion	120k	128	Sense2Vec	Pre-trained

### Multi-layer bidirectional transformer encoder: BERT

**Fig. 4:**
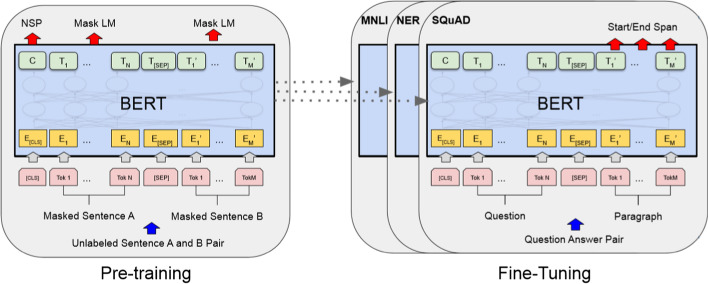
BERT pre-training and fine-tuning architecture overview. *Source* [60]

The use of word representations from pre-trained unsupervised methods is a crucial step in NER pipelines. Previous models such as Word2Vec [50], Glove [57], and FastText [54] focused on context-independent word representations or word embeddings. However, in the last few years models focused on learning context-dependent word representations, such as ELMo [58], CoVe [59], and the state-of-the-art BERT model [60], and then fine-tune these pre-trained models on downstream tasks. BERT is a context-dependent word representation model that is based on a masked language model and pre-trained using the transformer architecture [60]. BERT replaces the sequential nature of language modeling. Previous models such as RNN (LSTM and GRU) combines two unidirectional layers (i.e., Bi-LSTM), as a replacement for the sequential approach the BERT model employs a much faster attention-based approach. BERT is pre-trained in two unsupervised “artificial” tasks: (i) masked language modeling that predicts randomly masked words in a sequence, and hence can be used for learning bidirectional representations by jointly conditioning on both left and right contexts in all layers and (ii) next sentence prediction in order to train a model that understands sentence relationships. The transformer layer has two sub-layers: a multi-head self-attention mechanism, and a position-wise fully connected feed-forward network, followed by a normalization layer. Even though BERT learns a lot about language through pre-training it is possible to adapt the model by adding a customized layer on top of BERT outputs and then new training is done with specific data (this phase is called fine-tuning). We refer readers [60] for a more detailed description of BERT. An overview of the BERT architecture can be seen in Fig. 4.

Due to the benefits of the BERT model, we adopted the multilingual cased [60], the BETO [61] and the Biomedical language representation (BioBERT-Large) [62] pre-trained BERT models. Moreover, we trained from the scratch a Biomedical Spanish model (SBC-BERT) with 12 transformer layers (12-layer, 768-hidden, 12-heads, 110Mparameters) and a SoftMax output layer to perform the NER task. First, we replace the WordPiece tokenizer with the SentencePiece implementation [63] and the Spacy [32] tokenizer for sentence and subword segmentation. We train with a batch size of 128 sequences for 1,000,000 steps, which is approximately 40 epochs over the 4 million word corpus. We use Adam with learning rate of 1e$-$4. We use a dropout probability of 0.15 on all layers and a gelu activation function. Training of SBC-BERT was performed on 1 Cloud TPU, 8vCPUs Intel(R) Xeon(R) CPU @ 2.30 GHz and 16 GB memory. Details of train and pre-trained models can be seen in Table 23.

**Table 22:** Contextualized word models details

Detail	SBC-BERT	Bert-base-multilingual-cased	BETO cased	BioBERT-Large
Language	Spanish	104 languages	Spanish	English
Domain	Biomedical	General	General	Biomedical
Type	Contextual Word	Contextual Word	Contextual Word	Contextual Word
Corpus size	6 billion	3300M	3 billion	21.3 billion
Vocab size	200k	120k	31k	59k
Hidden size	768	768	1024	768
Algorithm	BERT train	BERT train	BERT train	BERT train
Property	Own	Pre-trained	Pre-trained	Pre-trained

### Datasets

We evaluate our deep learning approaches on the PharmaCoNER and the COVID-19 Open Research Dataset (CORD-19) datasets. The PharmacoNER dataset is a manually annotated corpus of 1000 clinical cases written in Spanish and annotated with mentions of chemical compounds, drugs, genes, and proteins. The dataset consists of Normalizables (4398), No Normalizables (50), Proteins (3009), and Unclear (167) labels. Further details can be found in [8].

**Table 23:** PharmaCoNER subsets details

Dataset	Subset	Documents	Sentences	Entities
PharmaCoNER	Train	500	8036	3822
	Valid	250	3759	1926
	Test	3751	62,000	

The CORD-19 dataset consists of over 181,000 scholarly articles written in English about COVID-19, SARS-CoV-2, and related coronaviruses. The dataset is manually annotated with disorder (18,704), species (30,343), chemical or drugs (11,173), gene and protein (57,738), enzyme (1480), anatomy (10,373), biological process (7765), molecular function (1722), cellular component (1099), pathway (517) and microRNA (690) unique entities. Further details can be found in [24]. In order to compare PharmaCoNER results with CORD-19 results we only evaluate on disorder, chemical or drugs and gene and protein entities. To the best of our knowledge, the CORD-19 dataset has not been used in any NER task or challenge. Therefore, we randomly split the dataset in training, validation and test datasets. Details about the datasets can be found in Table 24.

**Table 24:** CORD-19 subsets details

Dataset	Subset	Documents	Sentences	Entities
CORD-19	Train	8030	4015	7375
	Valid	2032	1016	1802
	Test	2776	1388	2647

## Data Availability

The PharmaCoNER and CORD-19 datasets that support the findings of this study are available in: PharmaCoNER Datasets, https://temu.bsc.es/pharmaconer/index.php/datasets/ CORD-19 Data, https://www.kaggle.com/allen-institute-for-ai/CORD-19-research-challenge Pre-trained word and concept representations are available in: Pubmed and PMC - Biomedical natural language processing, http://evexdb.org/pmresources/vec-space-models/ FastText-SBWC and Fastext 2M - English word vectors, https://fasttext.cc/docs/en/english-vectors.html W2V-SBWC - Spanish Billion Word Corpus and Embeddings, https://crscardellino.github.io/SBWCE/ Scielo+Wiki cased - FastText Spanish Medical Embeddings, https://doi.org/10.5281/zenodo.2542721 Sense2vec Reddit, https://pypi.org/project/sense2vec/ Bert-base-multilingual-cased - Whole Word Masking Models, https://github.com/google-research/bert BETO cased - BETO: Spanish BERT, https://github.com/dccuchile/beto BioBERT large, https://github.com/dmis-lab/biobert FastText-SBC, SNOMED-SBC, SBC-BERT trained from scratch embeddings models, https://github.com/rmriveraz/PharmaCoNER

## Abbreviations

NER: Namede Entity Recogntion
Bi-LSTM: Bidirectional Long Short-Term Memory
CRF: Conditional Random Field
UMLS: Unified Medical Language System
PoS: Part of Speech
SBWC: Spanish Billion Word Corpus
SBC: Spanish Biomedical Corpus
DDI: Drug Drug Interaction
SVM: Support Vector Machine
BERT: Bidirectional Encoder Representations from Transformers
